# Case Series of Cancer-associated Retinopathy (CAR)

**DOI:** 10.7759/cureus.4872

**Published:** 2019-06-10

**Authors:** Srikanth Naramala, Jowairiyya Ahmad, Sreedhar Adapa, Frank Gavini, Venu Madhav Konala

**Affiliations:** 1 Rheumatology, Adventist Medical Center, Hanford, USA; 2 Rheumatology, Orange Regional Medical Center, Middletown, USA; 3 Nephrology, The Nephrology Group, Visalia, USA; 4 Cardiology, Adventist Medical Center, Hanford, USA; 5 Internal Medicine/ Hematology and Oncology, Ashland Bellefonte Cancer Center, Ashland, USA

**Keywords:** retinopathy, cancer, paraneoplastic

## Abstract

Cancer-associated retinopathy (CAR) is a rare paraneoplastic disorder of the retina leading to blindness, associated with multiple cancers. It can lead to rapid progressive visual deterioration with retinal pathology ranging from retinitis pigmentosa to retinal degeneration. It is caused by antibodies directed against retinal antigens. This uncommon syndrome is a remote effect, independent of the primary tumor or metastatic lesion. We describe two cases of CAR, as well as pathophysiology, clinical manifestation, diagnostic criteria, and treatment of cancer-associated retinopathy.

## Introduction

Cancer-associated retinopathy (CAR) is a rare paraneoplastic disorder of the retina. It is associated with multiple cancers. It can present with sudden, progressive loss of vision associated with photosensitivity. Other findings include ring scotoma, attenuated retinal arteriole, visual field defects, abnormal electroretinogram (ERG), and the presence of circulating serum autoantibodies specific to retinal antigens.

This uncommon syndrome is a remote effect, independent of the primary tumor or metastatic lesion. It is heterogeneous with a wide variety of different ocular symptoms.

## Case presentation

Case 1

A 39-year-old African American female presented to the clinic complaining of severe fatigue and increased urinary frequency. She had a past medical history of systemic lupus erythematosus - diagnosed based on arthritis, discoid rash, positive antinuclear antibody (ANA), ribonucleoprotein antibody (RNP), anti-Ro (SS-A) antibody, and controlled on hydroxychloroquine (Plaquenil). Further evaluation revealed a normal complete blood count (CBC) with differential, complete metabolic panel, erythrocyte sedimentation rate (ESR), C-reactive protein (CRP), C3, C4, and double-stranded deoxyribonucleic acid (DNA). Urinalysis showed 2+ blood, along with red blood cells, and was negative for casts. She later underwent cystoscopy which revealed papillary urothelial carcinoma and subsequently underwent a left nephrectomy. Three years after her diagnosis of urothelial cancer, she presented with the sudden onset of visual blurring in both eyes. After an evaluation by a neuro-ophthalmologist, she was found to have constricted central and peripheral visual fields. Fluorescein angiography showed dense hyperfluorescence of the right eye and slight macular dystrophy, raising the concern for CAR. The genetic testing for mitochondrial disorders was negative. She had multiple positive anti-retinal antibodies which included anti-enolase, anti-GAPDH (glyceraldehyde 3-phosphate dehydrogenase), and anti-aldolase. Her treatment involved intravitreal triamcinolone injection in the right eye and she was concomitantly started on immunosuppressive therapy with prednisone and cyclosporine. There was no improvement in her vision with this therapy and it was subsequently switched to mycophenolate mofetil. She underwent a steroid implant six months later. Despite aggressive therapy, she had increased photosensitivity and was started on IVIG (intravenous immunoglobulin), complicated by hospitalization for aseptic meningitis. In the interim, she was under the care of urology and oncology for treatment of the bladder cancer with mitomycin.

Case 2

A 58-year-old African American female with a past medical history of hypertension, coronary artery disease, and diabetes mellitus type 2 presented with blurred vision. She described it as floaters and blackness in front of her eyes which later progressed to bilateral peripheral vision loss over a week's time. The ocular exam revealed extensive inflammatory findings in the anterior chamber, the posterior chamber, and the retinas bilaterally. She was placed on 1% prednisolone acetate eye drops and was referred to a retinal specialist for further evaluation of the abnormal retinal findings. The dilated fundoscopic exam revealed bilateral panuveitis, vitritis, retinal sheathing, and attenuation of vessels which was concerning for retinal vasculitis (Table 1). An autoimmune workup, including HLAB27, ANA, antineutrophil cytoplasmic antibody (ANCA), antiphospholipid antibody panel, serum angiotensin-converting enzyme (ACE), and rheumatoid factor (RF), was negative. An infectious disease workup, including human immunodeficiency virus (HIV), Lyme antibody titer, cytomegalovirus (CMV) antibodies, herpes simplex virus (HSV) antibodies, hepatitis serology, toxoplasmosis antibodies, Bartonella antibodies, syphilis rapid plasma reagin (RPR), and QuantiFERON-TB Gold Plus (Qiagen NV, Venlo, The Netherlands), were negative. Magnetic resonance imaging (MRI) of brain/orbits with contrast, as well as computed tomography (CT) of the chest, was non-revealing for significant pathology. She also had a history of chronic vaginal bleeding and back pain. Further examination revealed a pelvic mass, and a biopsy of the mass revealed endometrial carcinoma. This raised suspicion for CAR and the patient was tested for anti-retinal antibodies. She tested positive for anti-recoverin autoantibodies. She was initially treated with pulse dose steroids and referred for further management of her endometrial cancer.

**Table 1:** Slit-lamp Examination

OCULUS DEXTER (RIGHT EYE)	OCULUS SINISTER (LEFT EYE)
Intraocular pressure 14 mm Hg	Intraocular pressure 16 mm Hg
Pupil equal and reacting to light	Pupil equal and reacting to light
No afferent pupillary defect	No afferent pupillary defect
Conjunctiva: white and quiet	Conjunctiva: white and quiet
Cornea: normal	Cornea: normal
Iris: normal	Iris: normal
Anterior chamber: deep, 1+ cell flare	Anterior chamber: deep, 1+ cell flare
Lens: trace nuclear cataracts	Lens: trace nuclear cataracts
Vitreous: 2+ vitritis	Vitreous: 2+ vitritis
Optic nerve: flat, sharp, good color	Optic nerve: flat, sharp, good color
Macula: blunted	Macula: blunted
Retinal vessels: retinal sheathing, attenuated vessels 360 ° from the posterior pole to the periphery	Retinal vessels: retinal sheathing, attenuated vessels 360 ° from the posterior pole to the periphery

## Discussion

CAR is a rare paraneoplastic disorder of the retina leading to blindness and is associated with multiple cancers. It can lead to rapid progressive visual deterioration with retinal pathology ranging from retinitis pigmentosa to retinal degeneration. It is caused by antibodies directed against retinal antigens which include anti-recoverin, anti-α-enolase, anti-carbonic anhydrase II, heat shock cognate protein 70 (HSC70), anti-transducin-α autoantibodies, and anti-GADPH found months to years before cancer detection [1]. It was first described in 1976 by Sawyer et al. as vision loss and photoreceptor dysfunction [2].

It is a subtype of paraneoplastic visual syndromes which consists of four entities: CAR, melanoma-associated retinopathy (MAR), paraneoplastic optic neuropathy (PON), and bilateral diffuse uveal melanocytic proliferation (BDUMP). In this review, we will focus on the pathophysiology, clinical manifestation, diagnostic criteria, and treatment of CAR.

Breast cancer, small-cell lung cancer, gynecological, and hematological malignancies are the most common tumors associated with CAR. Other less frequent cancers associated with CAR are hepatocellular carcinoma, thymoma, prostate cancer, and colon cancer. At least in 50% of the patients, the diagnosis of CAR precedes a cancer diagnosis (which also happened in our second patient) [3-5].

In the case series reported by Adamus involving 209 patients, women are more affected than men and the age group affected is 40 - 85 years [3]. Both cases described in our article are female patients. The onset of retinopathy from a cancer diagnosis can vary from weeks to months (lymphoma and lung cancer) to years (breast cancer and prostate cancer). The diagnosis may be preceded by the presence of anti-retinal antibodies [3].

Tumor antigens trigger an immune response which results in the development of autoantibodies that cross-react with a retinal protein that leads to retinal degeneration and cell death. The mechanism by which anti-retinal antibodies target retinal antigens causing photoreceptor damage is not entirely understood [6]. The postulated molecular mechanism is apoptotic death of photoreceptors mediated by caspase-dependent pathways, along with intracellular calcium influx [7-8].

The diagnosis of CAR is based on the signs and symptoms, as well as the diagnosis of systemic cancer. There are no standard diagnostic criteria for CAR. The diagnosis involves the detection of anti-retinal antibodies, electrophysiologic evidence of retinal degeneration (such as attenuated retinal vessels), degenerative and atrophic changes of the retinal pigment epithelium, and optic disc pallor with concurrent clinical manifestations [4, 9].

The clinical phenotype involves sudden, progressive, bilateral, painless visual deterioration with an acute, subacute, and rarely, chronic presentation. There is degeneration of the cones and rods with symptoms of photoaversion, prolonged glare after light exposure, reduced visual acuity, decreased color perception, and central scotoma. On ocular exam, the fundus usually appears normal; occasionally, some patients may show vascular attenuation, optic disc pallor, and retinal pigment abnormalities [4, 9].

ERG findings involve abnormal rods and cones. A multifocal and full-field electroretinogram is used, with distinct changes in the ERG. ERG is more sensitive than optical coherence tomography (OCT) which may only illustrate mild findings in early disease. Abnormalities of a and b waves may be evident on ERG. Other findings on ERG include arterial attenuation, arteriolar sheathing, and periphlebitis. Optic disc pallor is seen in late cases. Slit-lamp examination rarely shows cellular debris in the anterior vitreous associated with low-grade inflammation in certain stages. Sawyer et al. showed there was photoreceptor degeneration of rods and cones with melanophages scattered in the outer retina and sparing of the ganglion cells in the inner retinal layer [2]. On OCT, diagnostic changes for pathologic anti-retinal antibodies include the loss of the outer retinal layer, such as the ellipsoid layer, and often show cystic spaces or occasionally mild schisis-like changes [7].

Greater than 50% of the patients with CAR will have serum autoantibodies that target retinal antigens. These antibodies have also been described in healthy individuals, although the appropriateness of the control sera in the study has been questioned [3, 7, 10]. In our patients, Case 1 was positive for anti-enolase, anti-GAPDH, and anti-aldolase antibodies. Case 2 was positive for anti-recoverin autoantibodies. 

There are a variety of lab techniques to detect these antibodies which include immunohistochemistry (IHC), Western blot, and enzyme-linked immunosorbent assay (ELISA). By using the Western blot technique, the serum from a CAR patient reacts with recoverin. Recoverin is a 23-kDa calcium-binding protein found on photoreceptors. It leads to the hypothesis that CAR is secondary to antibodies against tumor expressed recoverin that cross-reacts with photoreceptors [3, 7, 10].

The treatment of CAR involves treatment with corticosteroids associated with mild to moderate improvement in visual function based on anecdotal cases reports [4, 11]. Given the pathophysiology of the autoimmune process, early institution of immunosuppressive therapy appears to improve the chance of treatment response. A review of the literature shows that patients had responses to IV immunoglobulin [12], alemtuzumab [13], rituximab [14], and plasmapheresis [15]. Plasmapheresis is hypothesized to remove anti-retinal antibodies, circulating immune complexes, and cytokines which are contributing to the immunological response. However, since plasmapheresis is performed in combination with steroids, immunosuppressive medications response cannot be attributed entirely to plasmapheresis itself. Most recently, a naturally-occurring immunomodulator, Tolpa Torf preparation (Torf Corp. Pharmaceuticals Ltd., Wrocławskie, Poland), was found to be effective in reducing antibody levels [16].

Despite the above treatments, the visual prognosis remains poor. Treatment of the underlying cancer is unlikely to affect any visual prognosis. Overall survival depends on the underlying tumor and staging at diagnosis, as well as treatment options available [17].

## Conclusions

CAR is paraneoplastic retinopathy which might develop before cancer becomes clinically evident.

Screening for anti-retinal antibodies should be considered as part of the diagnostic workup in patients presenting with retinopathy with high pre-test probability. If paraneoplastic anti-retinal antibodies are detected in the absence of a known cancer, workup for an occult neoplasm is appropriate.

There is no epidemiologic data available on CAR because it is infrequently encountered in clinical practice. There are also no guidelines for the treatment of CAR. Treatment mainly involves corticosteroids, immunosuppressive therapy, and IV immunoglobulin. Alemtuzumab, rituximab, and plasmapheresis can be considered in refractory cases.
